# Applications of artificial intelligence in chronic kidney disease: a systematic review

**DOI:** 10.11606/s1518-8787.2026060007308

**Published:** 2026-09-02

**Authors:** Jalila Andréa Sampaio Bittencourt, Aline Santana Figueredo, Naruna Aritana Costa Melo, Cindy Lima Pereira, Yuri Armin Crispim de Moraes, Margareth Santos Costa Penha, Darah de Lourdes Costa Lindoso Marques, Evelyn Feitosa Rodrigues, Arthur André Castro da Costa, Allan Kardec Duailibe Barros

**Affiliations:** I Universidade Federal do Maranhão Rede Nordeste de Biotecnologia São Luís MA Brasil Universidade Federal do Maranhão. Rede Nordeste de Biotecnologia. São Luís, MA, Brasil; II Universidade Federal do Maranhão Departamento de Enfermagem Imperatriz MA Brasil Universidade Federal do Maranhão. Departamento de Enfermagem. Imperatriz, MA, Brasil; III Universidade Federal do Maranhão Rede Nordeste de Formação em Saúde da Família São Luís MA Brasil Universidade Federal do Maranhão. Rede Nordeste de Formação em Saúde da Família. São Luís, MA, Brasil; IV Universidade Federal do Maranhão Departamento de Farmácia São Luís MA Brasil Universidade Federal do Maranhão. Departamento de Farmácia. São Luís, MA, Brasil; V Universidade Federal do Maranhão Programa de Pós-Graduação em Saúde do Adulto São Luís MA Brasil Universidade Federal do Maranhão. Programa de Pós-Graduação em Saúde do Adulto. São Luís, MA, Brasil; VI Universidade Federal do Maranhão Departamento de Educação Física São Luís MA Brasil Universidade Federal do Maranhão. Departamento de Educação Física. São Luís, MA, Brasil; VII Faculdade Unibras Gama Departamento de Medicina Santa Inês MA Brasil Faculdade Unibras Gama. Departamento de Medicina. Santa Inês, MA, Brasil

**Keywords:** Artificial Intelligence, Machine Learning, Renal Insufficiency, Chronic, Systematic Review

## Abstract

**OBJECTIVE:**

To identify the applications of artificial intelligence in the early diagnosis, prediction and management of chronic kidney disease, highlighting the algorithms used, clinical outcomes, and impacts on nephrology practice.

**METHODS:**

In this systematic review, PubMed and Scopus databases were searched without year or language restrictions, including studies published between 2019 and 2025. Article selection followed PRISMA 2020 criteria, considering patients at risk for or diagnosed with chronic kidney disease, artificial intelligence tools as interventions, and outcomes related to early detection and disease management. Data was extracted on algorithm type, sample size, clinical outcomes, and main findings.

**RESULTS:**

Twenty-six studies were included, demonstrating the predominance of supervised learning and deep learning algorithms, generally showing high-performance metrics such as accuracy, sensitivity, and specificity in identifying chronic kidney disease and its stages. However, recurring weaknesses were observed, especially regarding the control of confounding factors, external validation of models, and standardization of datasets. Critical analysis of the studies shows that, despite the promising potential of computational models in the management of chronic kidney disease, methodological gaps still exist that limit their generalization and incorporation into clinical practice.

**CONCLUSION:**

This review demonstrates that artificial intelligence is a strategic tool for prevention, early diagnosis, and individualized management of chronic kidney disease. However, progress in this area depends on the development of more robust models, externally validated and integrated into real clinical contexts, contributing safely and effectively to renal health care.

## INTRODUCTION

Chronic kidney disease (CKD) is a progressive condition characterized by a reduction in the glomerular filtration rate (GFR) and/or the presence of kidney damage for a period of more than three months^1^. It is a highly prevalent and, in most cases, a silent condition, considered a major public health issue globally^2,3^.

Estimates indicate that more than 800 million people are affected by kidney disease worldwide, with CKD accounting for a significant portion of the morbidity and mortality associated with these conditions. Also, it is projected to become the fifth leading cause of death worldwide by 2040^3,4^. In Brazil, data from the *Sociedade Brasileira de Nefrologia* (Brazilian Society of Nephrology) indicate that more than 150,000 patients are undergoing renal replacement therapy, reflecting a growing demand for specialized services^5^.

Despite technological and scientific advances in the CKD diagnosis and treatment, significant gaps remain in early recognition of the disease and prediction of its progression, thus compromising the effectiveness of clinical interventions. One of the factors that exacerbates this scenario is the insidious nature of CKD: its most evident clinical manifestations generally appear only in advanced stages, when renal function is already significantly compromised^6,7^.

Traditional tools used in clinical practices (such as serum creatinine measurement, proteinuria detection, and GFR estimation equations) present limitations regarding sensitivity and in their ability to reflect the complexity of renal pathophysiology^4,8^. Therefore, it is essential to develop more robust approaches capable of integrating multiple clinical, laboratory, and demographic variables to provide more accurate and predictive analyses of renal health status^9^.

In this scenario, data analysis techniques based on artificial intelligence (AI) emerge as promising tools capable of providing more accurate and personalized predictions. The use of AI presents itself as a strategic tool for the development and improvement of screening, diagnostic, and clinical decision-making methods^9^.

Among these technologies, machine learning and artificial neural networks (ANN) stand out as innovative approaches in the healthcare field. These techniques enable the identification of complex patterns in large data sets, enabling the construction of predictive models that significantly contribute to risk stratification, personalized care, and treatment optimization^9,11,12^. AI applications are already widely recognized in specialties such as oncology, cardiology, endocrinology, and diagnostic imaging^13,14^.

In the field of nephrology, recent studies have explored the potential of AI and machine learning algorithms to estimate GFR, predict CKD progression, identify patients at higher risk of mortality, anticipate hospitalizations, and support individualized clinical management^3,9,11^. ANN-based models, in particular, demonstrate superior performance to traditional methods in several applications, especially due to their ability to handle nonlinear variables, complex interactions, and missing or incomplete data^15,16^.

Thus, the integration of AI tools into clinical practice represents a promising frontier for early diagnosis, offering new methods for predicting and mitigate the impact of kidney disease, especially in its early stages. Furthermore, AI can open up treatments and pathways for new preventive measures and personalized care plans for CKD management^3,17^.

Given the rapid growth in scientific production on this topic, it is necessary to systematize the existing evidence by identifying the main algorithms employed, the most frequently investigated outcomes, the databases used, and the potential impacts of these technologies on clinical practice. Therefore, this article aims to identify the applications of AI in the diagnosis, prediction, and management of CKD, highlighting the algorithms used, clinical outcomes, and impacts on nephrology practice.

## METHODS

This study is a systematic review based on the methodological guidelines for developing systematic reviews and meta-analyses of randomized clinical trials. This review has been registered in the Open Science Framework and is currently under embargo, awaiting automatic release of the DOI at the end of this period^18^.

The research was guided by the following guiding question: “The use of AI tools in patients at risk of or diagnosed with CKD is effective in the early detection, prediction, and management of the disease?” The research was constructed according to the PICO strategy (patient, intervention [factor and/or exposure and/or test being evaluated], comparison, and outcome); combining the characteristics of the patients (patients at risk or diagnosed with CKD), intervention (AI tools), comparison (conventional monitoring and prevention approaches), and outcome (early detection, prevention of progression, and disease management).

### Literature Search

The literature study search was conducted in the PubMed and Scopus databases, during July and August 2025, with no restrictions on year or language for the selected studies. Boolean operators AND and OR were used to combine controlled descriptors, according to the following search strategy:

(“machine learning” AND “chronic kidney disease”) OR (“machine learning” AND “artificial intelligence”) OR (“machine learning” AND “chronic kidney disease” AND “neural networks” AND “artificial intelligence”).

Studies obtained in all combinations and databases were organized and analyzed using a free, single-version, and web-based review platform called Rayyan Qatar Computing Research Institute (Rayyan QCRI)^19^. Initially, duplicate removal was performed, followed by the preliminary selection and the eligibility assessment.

### Study Selection

**Figure 1:**
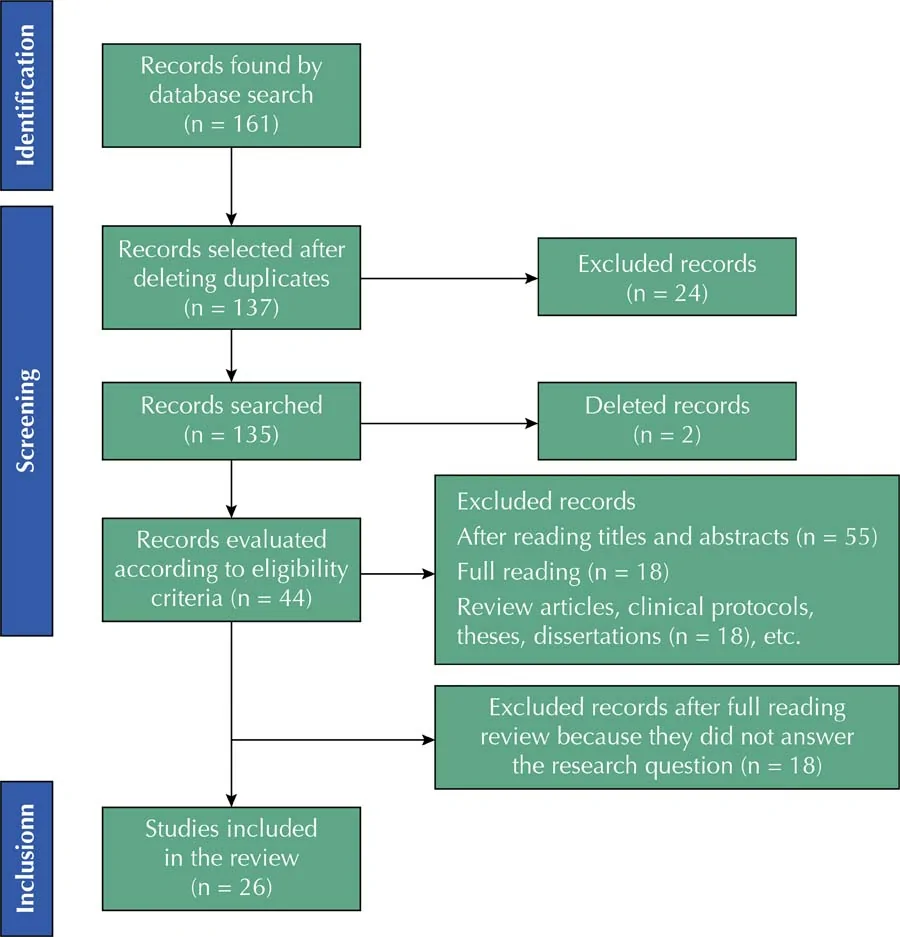
Identification of studies through databases, according to PRISMA recommendation.

The study selection process was conducted independently by two reviewers (ASF and JASB), and disagreements were resolved by a third one (NACM). In the first stage, the titles of the identified documents through the search strategy were analyzed, and potentially eligible studies were pre-selected. In the second stage, the abstracts were read, and subsequently the selected texts were analyzed in full for decision regarding inclusion or exclusion from the review (Figure 1).

### Data Extraction

The included studies had their data extracted by a reviewer (CLP), using a form adapted from the Cochrane Development, Psychosocial and Learning Problems (Cochrane). Also, this extracted data were recorded in a Microsoft Excel spreadsheet, and the extracted variables were: author, year, country, sample size, AI technique, clinical outcomes, and main findings.

### Risk of Bias

The methodological quality analysis of the studies was performed following the Joanna Briggs Institute Checklist for Prevalence Studies^20^. This instrument is composed of eight domains, which were adapted to evaluate the 26 studies in this review, namely: clearly defined inclusion criteria; adequate description of the population/sample; valid and reliable measurement of input variables; valid measurement of the outcome (CKD, progression, stage, etc.); identification and control of confounding factors; appropriate statistical analysis/model validation; use of external validation or independent set; clinical applicability and transparency of the model.

For each study, the responses to the eight domains were classified as “yes” or “no,” with positive responses (“yes”) indicating better methodological quality and a lower risk of bias. The final classification of the risk of bias was based on the proportion of “yes” responses obtained in each study. Studies that presented up to 49% “yes” responses were categorized as high risk of bias; those with 50% to 69%, as moderate risk; and those with 70% or more, as having a low risk of bias^20^.

## RESULTS

### Description of the Studies

This review was conducted based on the recommendations of the Preferred Reporting Items for Systematic Reviews and Meta-Analyses (PRISMA) 2020 statement for reporting systematic reviews and meta-analyses of randomized controlled trials^21^.

Therefore, this review consists of 26 articles published between 2019 and 2025. A continuous increase was observed over the years, with 2025 having the highest number of publications (n = 8), followed by 2023 (n = 5), 2021 and 2022 (n = 4 each), and 2024 (n = 3). 2019 and 2020 showed only one publication each (n = 1) (Chart 1).

**Chart 1:** Methodological design of the studies selected for the systematic review, 2025.

Author (year)	Study type	Data sources	Nature of the data
Peng et al.^22^ (2021)	Retrospective	Hospital Cohort	Clinical and laboratory data
Lee et al.^23^ (2022)	Retrospective	University Hospital	Images (Ultrasound) + clinical data
Daniel et al.^24^ (2021)	Technical Validation Study	Imaging Center	Images (MRI)
Abdel-Fattah et al.^25^ (2022)	Retrospective	Clinical Big Data (Apache Spark)	Clinical and laboratory data
Neela et al.^26^ (2025)	Computational Study	Image Databases	Images (CT scan)
Pechprasarn et al.^27^ (2025)	Retrospective Study	UCI Dataset	Clinical and laboratory data
Khan et al.^28^ (2021)	Simulation	Public Database	Clinical and laboratory data
Chowdhury et al. ^29^ (2025)	Retrospective Study	Cohort with diabetic patients	Clinical and laboratory data
Norouzi e Kahriman^30^ (2024)	Methodological Study	Public Dataset	Clinical and laboratory data
Al-Rasheed et al.^31^ (2025)	Retrospective Study	Public Dataset	Clinical and laboratory data
Rubia et al.^32^ (2024)	Retrospective Cohort	Electronic Health Records	Clinical and laboratory data
Takkavatakarn et al.^33^ (2023)	Retrospective Study	University Hospital Data Sources	Images (USG)
Kuo et al.^34^ (2019)	Retrospective Study	Clinical Dataset	Clinical and laboratory data
Singamsetty et al.^35^ (2024)	In Silico Study	Pharmacometric Simulation	Simulated time series
Gaweda et al.^36^ (2022)	Retrospective Study	Clinical Dataset	Clinical and laboratory data
Al-Momani et al.^37^ (2022)	Comparative Study	Clinical Dataset	Clinical and laboratory data
Sawhney et al.^38^ (2023)	Retrospective Cohort	National EHR (TriNetX)	Electronic medical records
Zhang et al.^39^ (2025)	Computational Study	Image Database	Renal images
Kumar et al.^40^ (2023)	Methodological Study	Clinical Dataset	Clinical and laboratory data
Sharma et al.^41^ (2021)	Methodological Study	Clinical Dataset	Clinical and laboratory data
Alikhan et al.^42^ (2023)	Computational Study	Health Big Data	Heterogeneous clinical data
Nazari e Abdelrasoul^43^ (2025)	Retrospective Study	Hospital cohort	Clinical and demographic data
Liu et al.^44^ (2025)	Methodological Study	Renal Biopsies	Histopathological images
Sharma et al.^45^ (2025)	Retrospective Study	Clinical Dataset	Clinical and laboratory data
Pareek et al.^46^ (2023)	Computational Study	Clinical Dataset	Clinical and laboratory data
Lokuarachchi et al.^47^ (2020)	Cross-sectional Study	Community Cohort	Clinical and laboratory data

CNN: convolutional neural network; SVM: support vector machine; ANN: artificial neural network; KNN: K-nearest neighbors; DNN: deep neural network; RNN: recurrent neural network.

Regarding geographic distribution, India had the highest number of publications, with seven studies (n = 7; 25.9%), followed by the USA (n = 6), and China (n = 5). The other countries contributed sporadically, including Saudi Arabia (n = 1) and Egypt, Thailand, Bangladesh, Iraq, Taiwan, Sri Lanka, and South Korea (n = 1 each). International collaborations, such as the Pakistan–Saudi Arabia–Egypt and Saudi Arabia–India–Czech Republic collaborations, also stand out, highlighting the global and collaborative nature of the research in the area.

### Types of Data Used

When analyzing the studies included in this review, a predominance of retrospective studies based on clinical laboratory data was observed. Most of them are applications of AI in CKD. It was also observed that more than 60% of the articles used structured clinical databases, originating from hospital records or public datasets, focusing on early prediction, risk stratification, and disease progression^22^.

It was also observed that a significant number of studies used large-scale electronic health records, mainly population studies that used time series, allowing for long-term follow-up of patients, as well as the prediction of outcomes such as progression to end-stage renal disease and the need for replacement therapy^33,34,39^.

As for image-based studies, they represent an important approach, using magnetic resonance imaging, ultrasound, computed tomography, and histopathological slides for automated diagnosis and renal segmentation, especially through convolutional neural networks (CNN) and deep architectures^23,24,26,34,40,44,46^.

Furthermore, some studies also explored simulation and big data environments, including in silico models and distributed platforms, highlighting the potential of AI for complex applications in the management of CKD, although these studies had limitations regarding external clinical validation^28,36,42^.

### Algorithms Used and their Main Applications in CKD

The analysis of the AI techniques employed in the 26 selected articles revealed a predominance of supervised learning algorithms, notably Random Forest (28.6%), CNN (22.9%), and support vector machines (SVM) (20.0%). These methods have been widely applied to risk prediction, patient stratification, and medical image analysis. Classical techniques, such as ANN, decision trees, and logistic regression, also showed considerable frequency, reflecting their consolidation in clinical settings. Methods such as Naive Bayes and K-Nearest Neighbors (KNN) appeared in smaller proportions, generally associated with performance comparisons.

**Figure 2:**
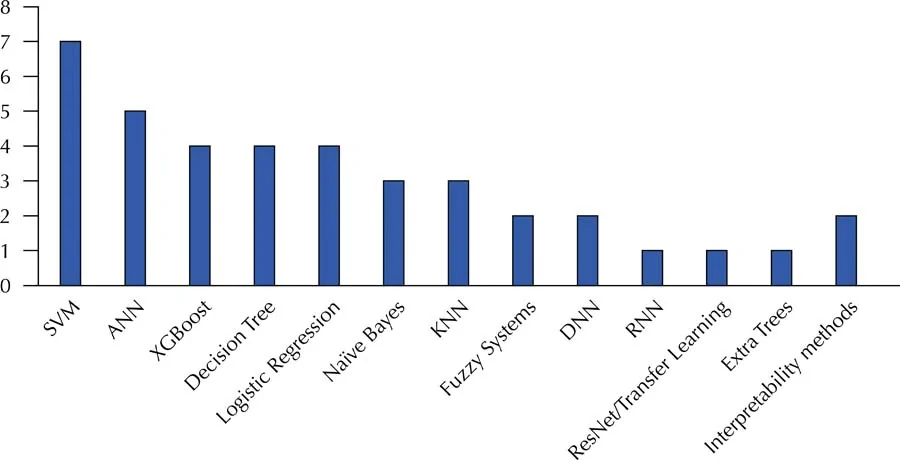
Main artificial intelligence used in the studies.

Among the most recent approaches, we observed the use of XGBoost and advanced deep learning architectures, including residual neural networks (ResNet) with transfer learning, deep neural networks (DNN), and recurrent neural networks, applied primarily to image and time series data. Furthermore, some publications incorporated interpretable AI techniques, such as Shapley Additive Explanations (SHAP) and Local Interpretable Model-agnostic Explanations (LIME), highlighting the concern for the transparency and clinical applicability of these models (Figure 2).

In the context of applying algorithms to CKD, there was greater application in early diagnosis and prediction of the disease through machine learning methods^22^. Thus, the most used models were Random Forest, SVM and ANN, used mainly in analyses of structured clinical and laboratory data, from hospital databases, observational studies and electronic medical records^22,25,28,33,37^.

Random Forest and SVM algorithms have been most applied in secondary prevention and initial diagnosis of CKD, demonstrating good performance in identifying individuals at risk from routine clinical and biochemical variables, such as serum creatinine, estimated GFR, and metabolic parameters^22,25,27,37,38^.

ANN, including multilayer perceptrons and DNN, have been employed for both diagnosis and prediction of CKD progression, highlighting applications related to disease stage stratification and identification of prognostic factors^22,28,31,35,37,38,45^. Some studies have advanced the use of explainable approaches, such as SHAP and TabNet, seeking to increase the interpretability of the models and facilitate their integration into clinical practice, especially in supporting decision-making^29,35,43^.

In the field of medical image analysis, there has been increasing use of CNN, mainly applied to the diagnosis and early detection of CKD through imaging exams, such as renal ultrasound, magnetic resonance imaging, and histopathological images^23,24,26,31,34,40,42,44,46^. In these studies, CNNs were used both for diagnostic classification and for automatic segmentation of renal structures, demonstrating potential to reduce dependence on the human evaluator and standardize the interpretation of exams^23,24,34-44^.

Deep learning-based models have also been employed in predicting CKD progression and the risk of developing end-stage renal disease, especially in studies that used large electronic health record databases^25,33,39^. Algorithms such as XGBoost and other ensemble methods have demonstrated good performance in population risk stratification, being applied mainly in clinical surveillance and health care planning contexts^33,39^.

To a lesser extent, some studies have explored alternative approaches, such as fuzzy logic, expert systems, and recurrent neural networks^28,36,47^. These methodologies have been primarily directed towards prevention, community screening, and clinical management of CKD, including applications aimed at longitudinal follow-up and treatment personalization, although they still have limited use and an experimental character^36,41,47^.

Considering this, it is noteworthy that the 26 articles analyzed indicate that AI stands out for its ability to improve diagnostic accuracy, facilitate risk stratification, and support the clinical management of CKD. Ensemble models such as Random Forest and XGBoost, as well as advanced deep learning techniques, demonstrated superior performance to conventional methods. This confirms the potential of AI as a strategic tool for early detection, progression monitoring, and clinical decision-making support in patients at risk for or diagnosed with CKD (Chart 2).

**Chart 2:** Characteristics of the studies included in this systematic review, 2025.

Author (year)	Sample size	Main objective of the article
Peng et al.^22^ (2021)	400 patients	To develop a two-stage predictive model based on neural networks to identify and predict the occurrence of chronic kidney disease (CKD) more accurately.
Lee et al.^23^ (2022)	909 patients	To propose a machine learning-assisted CKD diagnostic system using renal ultrasound images combined with computationally extracted quantitative features.
Daniel et al.^24^ (2021)	116 subjects	To develop and validate a convolutional neural network for automatic segmentation of kidneys in magnetic resonance imaging of healthy individuals and those with CKD.
Abdel-Fattah et al.^25^ (2022)	400 patients	To predict the presence of CKD using a hybrid machine learning model implemented in an Apache Spark environment, aiming for scalability and computational efficiency.
Neela et al.^26^ (2025)	Not provided	To predict the progression of CKD using models based on convolutional neural networks (CNNs), focusing on monitoring the evolutionary progression of the disease.
Pechprasarn et al.^27^ (2025)	200 patients	Optimize CKD prediction using machine learning models with a minimal set of diagnostic predictors, reducing costs and clinical complexity.
Khan et al.^28^ (2021)	25 patients	Model, simulate, and optimize intelligent kidney disease prediction systems using computational intelligence techniques.
Chowdhury et al.^29^ (2025)	Not provided	Develop a deep learning-based model (TabNet) for early detection of CKD stages in diabetic patients.
Norouzi e Kahriman^30^ (2024)	Not provided	Propose an early CKD diagnostic model using machine learning algorithms, with emphasis on the SPEGASOS method.
Al-Rasheed et al.^31^ (2025)	Not provided	Classify kidney diseases using a deep learning model with dense layers, aiming for greater diagnostic accuracy.
Rubia et al.^32^ (2024)	Not provided	Develop an automatic kidney disease prediction system based on deep learning techniques.
Takkavatakarn et al.^33^ (2023)	3,160 patients	Develop and evaluate machine learning models capable of predicting progression to end-stage renal disease in patients with stage 4 CKD.
Kuo et al.^34^ (2019)	Not provided	Automate the prediction and classification of renal function using ultrasound images analyzed by deep learning models.
Singamsetty et al.^35^ (2024)	Not provided	To improve the predictive capacity of machine learning models for CKD by incorporating explainable artificial intelligence (XAI) techniques.
Gaweda et al.^36^ (2022)	80 patients	To apply artificial intelligence to guide precision treatment of CKD associated with mineral-bone disorders.
Al-Momani et al.^37^ (2022)	400 patients	To detect the presence of CKD using machine learning techniques applied to clinical data.
Sawhney et al.^38^ (2023)	400 patients	To compare different artificial intelligence models used in the early prediction and assessment of CKD.
Zhang et al.^39^ (2025)	104,565 patients	To predict acute and chronic kidney disease in the post-Covid-19 period using machine learning models applied to US national electronic health records.
Kumar et al.^40^ (2023)	Not provided	To recognize and predict kidney disease using deep learning techniques associated with medical image processing.
Sharma et al.^41^ (2021)	Not provided	To use fuzzy logic as a tool for predicting kidney disease from clinical variables.
Alikhan et al.^42^ (2023)	Not provided	To diagnose CKD in big data systems using a CNN with a self-attention mechanism optimized by a seasonal optimization algorithm.
Nazari e Abdelrasoul^43^ (2025)	1,177 patients	Analyze clinical and demographic variables using neural networks to predict platelet count and prothrombin time in patients with CKD.
Liu et al.^44^ (2025)	Not provided	Develop a self-supervised learning framework for automatic segmentation of multiple tissues in renal histological images.
Sharma et al.^45^ (2025)	Not provided	Detect and predict CKD using machine learning algorithms applied to clinical data.
Pareek et al.^46^ (2023)	Not provided	Predict early-stage CKD using convolutional neural networks, focusing on early detection.
Lokuarachchi et al.^47^ (2020)	Not provided	Predict CKD of unknown etiology (CKDu) using neural networks based on quality of life score (KDQOL), ankle edema, and risk factors. Main Objective of the Article

CKD: chronic kidney disease.

### Risk of Bias in Studies

The methodological quality analysis of the studies in this review was performed based on the Joanna Briggs Institute domains^20^, considering aspects related to sample selection, measurement of variables and outcomes, control of confounding factors, analysis and validation of models, as well as clinical applicability.

**Figure 3:**
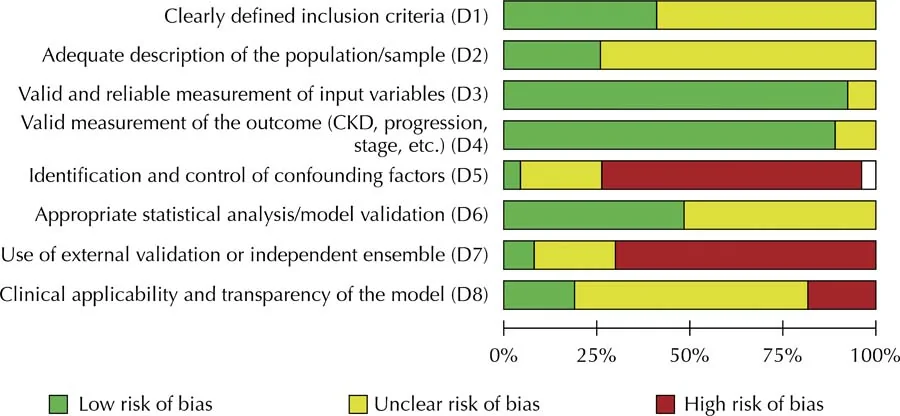
Risk of bias of the studies included in this systematic review using the Joanna Briggs Institute critical appraisal tool for prevalence studies.

In general, it was observed that more than 75% of the studies presented positive responses (“yes”) for the domains related to the definition of outcomes and the measurement of input variables, indicating a low risk of bias in these aspects. On the other hand, a low proportion of positive responses (“yes”) was found in the domains related to the control of confounders (> 25%) and the external validation of the models (> 25%), characterizing a moderate to high risk of bias in these items, which may limit the generalization and applicability of the findings (Figure 3).

## DISCUSSION

The CKD represents a serious global public health problem, characterized by high morbidity and mortality and high costs related to renal replacement therapy. In this scenario, AI has emerged as a promising tool for prevention, early diagnosis, and prediction of clinical outcomes. The integrated analysis of the twenty-seven studies included in this review highlights important advances, but this highlights challenges that need to be overcome.

Therefore, the results of this systematic review demonstrate convergence among the studies analyzed regarding the effectiveness of using AI techniques in the detection, prediction, and monitoring of CKD. Regardless of the type of algorithm employed, satisfactory performance of the models is observed, especially in early diagnosis and risk stratification, corroborating the findings reported by Khan et al.^28^ and Sawhney et al.^38^, which highlight the ability of these technologies to identify subclinical patterns not easily detectable by conventional methods.

Studies based on tabular clinical and laboratory data also presented consistent results, indicating that variables routinely available in clinical practice are sufficient to build robust predictive models. Works such as those by Norouzi and Kahriman^30^, Pechprasarn et al.^27^ and Sharma et al.^41^ demonstrated that reduced sets of predictors maintain high accuracy, a finding that directly relates to the results of this review, which point to the feasibility of applying these models in screening and primary care scenarios. This convergence suggests that simplifying the models does not necessarily compromise their performance but rather expands their implementation potential.

In contrast, studies that used medical images, such as ultrasound, magnetic resonance imaging, and histopathology, reported superior accuracy, especially in renal morphological segmentation and classification tasks^24,40,44^. These findings complement the results of the review by indicating that image-based models are particularly useful at secondary and tertiary levels of care, functioning as tools to support specialized diagnosis. However, the need for advanced technological infrastructure and large labeled databases reinforces the limitation observed in the results regarding the applicability of these models in resource-limited contexts^24,48^.

The integration of big data and hybrid models has also been explored. The use of high-performance platforms, such as Apache Spark, combined with supervised learning algorithms, has proven effective in predicting CKD in large cohorts, achieving superior performance when applying feature selection methods and hybrid models^25^.

Other studies focused on predicting CKD progression and clinical outcomes, such as evolution to end-stage renal disease, showed strong alignment with each other and with the results of the review. Takkavatakarn et al.^33^ and Chowdhury et al.^29^ showed that advanced models, such as DNN and attention-based architectures, are able to anticipate adverse outcomes with greater accuracy than traditional methods. These findings reinforce the potential of AI not only as a diagnostic tool, but also as a strategic instrument for therapeutic planning and the allocation of health resources^44,48,49^.

Another relevant point refers to the increasing incorporation of explainable AI. Since the interpretability of models improves clinical acceptance and confidence in the results, an aspect that directly relates to the findings of this review, which identified the “black box” as a recurring barrier to the adoption of these technologies. Explainability therefore emerges as a central element for the translation of research models into clinical practice^35,50^. Additional deep learning studies also corroborate this advance, achieving accuracy above 99% in CKD classifications in public databases^31^.

In the context of prevention and early detection, studies have shown that AI can identify individuals at risk of developing CKD or progressing to more severe stages early on using easily accessible variables such as demographic data, comorbidities, and diagnostic history^48,49^. Approaches using Bayesian networks and SVM in Japanese cohorts have also demonstrated the capacity to stratify risk, including in individuals classified as low clinical risk^50^. These findings reinforce the potential of AI in preventive screening programs, especially in low- and middle-income settings.

From a public health perspective, the findings of this review reinforce the potential of AI as a strategic tool for addressing CKD at different levels of care. The high accuracy of models based on routine clinical and laboratory data suggests the feasibility of application in primary care settings and in health systems with limited resources, contributing to population screening and early identification of individuals at risk^33,34,38,39^. This aspect is particularly relevant given the increasing burden of CKD and inequalities in access to specialized diagnosis. Furthermore, the integration of predictive models with electronic health records expands opportunities for epidemiological surveillance and large-scale clinical decision-making support^48^. Thus, although methodological and implementation challenges persist, the progressive incorporation of AI may promote more equitable, timely, and cost-effective strategies for managing CKD within health systems.

### Strengths and Limitations of the Study

Conducting a systematic review on the application of AI in the diagnosis, prediction, and management of CKD presents important strengths, especially given the exponential growth of studies using machine learning and deep learning techniques in the field of nephrology. One of the main strengths of this approach is the possibility of systematizing and integrating dispersed evidence, allowing a comprehensive view of the performance, clinical applications, and methodological trends of different AI models. This favors the identification of common patterns, knowledge gaps, and future directions for research and technological innovation.

Another positive aspect refers to the comparative evaluation between different algorithmic approaches, data types (clinical, laboratory, imaging, and electronic health records), and application contexts. By bringing together studies with different designs and objectives, the systematic review makes it possible to understand how AI has been used along the continuum of CKD, from early screening to outcome prediction and support for clinical management. In addition, this synthesis contributes to the translation of scientific knowledge into clinical practice, assisting health professionals and managers in making evidence-based decisions.

Therefore, it is noteworthy that this research is relevant for contributing to clinical practice. However, as a literature review, it has some limitations, such as methodological heterogeneity among studies, including differences in populations, variables used, algorithms applied, and metrics evaluated, thus hindering direct comparison of results^22^.

Furthermore, many studies were restricted to local cohorts, requiring prospective external validation across multiple centers. Ethical and scientific integrity issues also arise, as they were evidenced in articles subsequently retracted, reinforcing the need for methodological rigor and transparency^22^. Another critical point concerns the integration of these models into clinical practice, which requires not only high statistical performance but also evidence of their real impact on clinical outcomes, costs, and patient quality of life.

However, the analysis of the studies revealed that AI represents a strategic supporter in the prevention, early diagnosis, and management of CKD, with promising results on various fronts, from population risk stratification to personalized clinical approaches. Models based on deep learning, reinforcement learning, and hybrid algorithms have demonstrated superior performance to traditional techniques, suggesting the potential to transform nephrology practice by reducing costs, increasing diagnostic accuracy, and anticipating therapeutic decisions.

Furthermore, ethical, transparency, and interpretability issues remain key barriers to full acceptance by healthcare professionals. Therefore, it can be concluded that AI represents a promising frontier for nephrology, but its consolidation will depend on methodological rigor, multicenter validation, and a balance between high performance and explainability, ensuring real benefits for patients with CKD.

### Recommendations

Based on the findings of this systematic review, it is recommended that future research on the application of AI in the diagnosis, prediction, and management of CKD prioritize multicenter and prospective studies capable of evaluating the generalizability and robustness of the models in different epidemiological contexts and health systems.

It is also recommended to invest in methodological standardization, especially regarding the selection of variables, performance metrics, and validation strategies. The adoption of specific guidelines for AI studies applied to health, such as TRIPOD-AI, CONSORT-AI, and STARD-AI, can contribute to greater transparency, reproducibility, and comparability among studies, strengthening the quality of the evidence produced.

In the context of clinical practice, it is suggested that AI models be developed focusing on implementation feasibility, prioritizing the use of routinely available clinical and laboratory data, especially in primary care settings and in regions with limited resources. In parallel, the incorporation of explainable AI techniques is recommended to increase the confidence of healthcare professionals, facilitate the interpretation of results, and support shared clinical decision-making.

In addition, it is recommended that future research broaden the scope of the data used, integrating social, environmental, and behavioral factors, recognizing CKD as a multifactorial condition. This approach can contribute to models that are more sensitive to health inequalities and more aligned with the needs of vulnerable populations.
